# Seed yield can be explained by altered yield components in field-grown western wheatgrass (*Pascopyrum smithii Rydb*.)

**DOI:** 10.1038/s41598-019-54586-0

**Published:** 2019-11-29

**Authors:** Zhao Chen, Junpeng Niu, Xinlong Cao, Wenbo Jiang, Jian Cui, Quanzhen Wang, Quan Zhang

**Affiliations:** 1 College of Grassland Agriculture, Northwest A&F University, Yangling, 712100 Shaanxi Province China; 2 College of Life Science, Northwest A&F University, Yangling, 712100 Shaanxi Province China; 3 Jiuquan Daye Seed Industry Co. Ltd., Jiefang Road, #325, Suzhou Qu, 735000 Jiuquan, Gansu Province China

**Keywords:** Plant sciences, Plant breeding

## Abstract

Western wheatgrass (*Pascopyrum smithii* Rydb.) is an important cool-season forage and turfgrass. However, due to seed dormancy and poor seedling vigor, it is difficult to develop high seed yield production systems, and assessing these components in response to seed yield. Based on multifactor orthogonally designed field experimental plots under various field management regimes, the effects of numbers of fertile tillers m^−2^ (Y_1_), spikelets/fertile tiller (Y_2_), florets/spikelet (Y_3_), seed numbers/spikelet (Y_4_), and seed weight (Y_5_) on seed yield (Z) were determined over three successive years. Correlation analysis indicated that fertile tillers (Y_1_) was the most important seed yield component. And the biggest contribution of those five yield component is fertile tillers (Y_1_), followed by seed numbers/spikelet (Y_4_), spikelets/fertile tiller (Y_2_), florets/spikelet (Y_3_) and seed weight (Y_5_), respectively. By using ridge regression analysis, we have developed an accurate model of seed yield with its five components. Finally, the results of synergism and antagonism among these yield components on seed yield showed that fertile tillers and seed numbers/spikelet had an antagonistic effect on seed yield. Therefore, selection for high seed yield by direct selection for large values of fertile tillers and seed numbers/spikelet would be the most effective breeding strategy for western wheatgrass.

## Introduction

Western wheatgrass (*Pascopyrum smithii* Rydb.) is a native, perennial cool-season grass, found most abundantly in the southern, mixed-grass prairie region of the Great Plains of North America and is grown for livestock production throughout the temperate regions of the world^1^. Because it thrives on impoverished soils in pastoral environments, even with multiple, simultaneous stressors^2^, it is an important species for soil protection, water conservation, and vegetation protection in arid and semi-arid regions. Due to these characteristics, it is a competitive, high-yielding species, providing forage for livestock and wildlife on semi-arid rangelands in Eurasia and northwest China^3–6^. Thus, it can appear as a dense monoculture^7,8^. Seed yields in the cool-season perennial grasses are often low, and in China, the seed supply of perennial grasses has depended on imports for many years due to inadequate supplies of locally produced high quality seed. To become more self-sufficient in supplying seed for its many needs, the Chinese government has encouraged the development of increased seed production capacity^9,10^.

There have been many studies on the factors affecting crop yields, with the aim of improving yields as much as possible^11^. Seed yield is a complex trait that is the culmination of growth and developmental processes that are influenced by multiple yield components^12,13^. Understanding the relationship between yield and yield components and the correlations between yield components is a prerequisite for building an efficient breeding program^14^. To date, no research has been conducted to develop models for seed yield and related yield components in western wheatgrass. Therefore, it is necessary to examine the relationships among various factors, especially seed yield, yield components, and their interdependence. To create a high seed yield breeding program, a mathematical model needs to be developed that accurately predicts forage and seed yield, and this must subsequently be validated through field experiments^15^. Owing to the lower complexity and lower environmental influence of yield components compared to yield, the use of these traits to practice indirect selection for yield is justified^16^. Several research groups have attempted to determine the association between the characters of forage yield. To obtain a high-yield forage species, several scholars have focused their attention on the agronomic traits of yield groups, which are closely related to seed yield. Annapurna *et al*.^17^ found that seed yield showed a significant positive correlation with plant height, ear diameter, number of seeds per row, and number of rows per ear. Assefa *et al*.^18^ reported correlations between yield and planting density, and other yield components. Tang *et al*.^19^ found that grain yield per plant showed a highly significant positive correlation with the 1000-grain weight, plant height and planting density. Traits related to the generative parts of the plant, such as pods per plant, number of seeds per pod, number of fertile spikelets per panicle, panicle length, spikelet density, number of filled seeds, number of effective tillers per plant, and 1000 seed weight, are the most frequently considered parameters^20–25^. Thus, plant morphological traits such as fertile tillers m^−2^ (Y_1_), spikelets/fertile tillers (Y_2_), florets/spikelet (Y_3_), seed numbers/spikelet (Y_4_), or seed weight (Y_5_), can be valuable in defining the best criteria for selection in biological and agronomic studies.

Since seed yield is influenced by environmental conditions, and agronomic factors^26–28^, the experimenter selects the best design based on the information available with respect to field spatial heterogeneity^29^. Hexi Corridor, in Gansu Province, China, is considered a key seed production centre due to plentiful mountain run-off water, groundwater and dry, warm summer conditions. It is the largest maize seed production area and one of the main vegetable and flower seed production regions in China. Thus, we chose this area to evaluate the potential seed production of cool-season grasses. The orthogonal experimental design (OED) method allows researchers to test the effectiveness of many interventions simultaneously in a single experiment (and identify their interactions) with far fewer experimental units than it would take to exhaust all possible intervention combinations using other techniques. Therefore, since our objective was to investigate relationships among seed yield traits and develop a model of seed yield and yield components, we chose the OED. The aim of this study was to confirm the direct and indirect effects of key seed yield components, including fertile tillers m^−2^ (Y_1_), spikelets/fertile tillers (Y_2_), florets/spikelet (Y_3_), seed number/spikelet (Y_4_), and seed weight (mg) (Y_5_), on seed yield (Z) of *Pascopyrum smithii* based on a multifactor orthogonal design under field conditions, with various managements.

Formulae representing the theoretical relationship between the seed yield components and seed yield (or potential seed yield) are represented as: Z_SY_ = Y_1_ × Y_2_ × Y_3_ × Y_4_ × Y_5_. This study evaluated two hypotheses: (1) all five seed yield components and the seed yield are inter-related, and each yield factor has different direct and indirect effects on seed yield, and (2) an algorithmic model of seed yield based on these five components can be developed to accurately estimate seed yield.

## Results

### Correlations between traits

Pearson correlation analysis revealed that seed yield (Z) was significantly and positively influenced (*P* < 0.001) by seed yield components Y_1_ and Y_2_, but was negatively affected to a lesser extent by Y_4_ and Y_5_ (*P* < 0.05 or *P* < 0.01) (Table 1). Y_1_ had the maximum coefficient on Z (0.472, *P* < 0.001). The correlations among Y_1_ through Y_5_ showed significance (*P* < 0.001), while Y_1_ and Y_2_ were negatively correlated with Y_3_ and Y_5_. However, Y_1_ and Y_2_ showed strong, positive correlations with Z in for all three years (*P* < 0.001) (Table 2). The order of the strength of correlation coefficients in each year was 2004 < 2003 < 2005. Y_1_ had the strongest positive influence on Z (0.452, 0.534, and 0.657 for 2003, 2004 and 2005, respectively), and Y_5_ had the strongest negative influence on Z (−0.219, −0.209 and −0.354 for 2003, 2004 and 2005, respectively, Table 2).

**Table 1:** Matrix of Pearson correlation coefficients of Y_1_~Y_5_, Z (*Pascopyrum smithii* Schreb.) averaged over 3 years.

Seed yield components	Y_1_	Y_2_	Y_3_	Y_4_	Y_5_	Z(seed yield)
Y_1_	1.0000	0.118**	−0.056**	−0.071**	−0.286**	0.472**
Y_2_		1.0000	−0.055**	−0.051**	−0.104**	0.116**
Y_3_			1.0000	0.105**	0.155**	−0.023
Y_4_				1.0000	0.064**	−0.030*
Y_5_					1.0000	−0.192**

*Significant at the 0.05 probability level.

**Significant at the 0.01 probability level.

N = 380.

**Table 2:** Matrix of Pearson correlation coefficients of Y_1_~Y_5_, Z (*Pascopyrum smithii* Schreb.) for each year.

	year	Y_1_	Y_2_	Y_3_	Y_4_	Y_5_	Z
Y_1_	2003	1.0000	0.435**	−0.297**	−0.469**	−0.434**	0.534**
	2004	1.0000	0.127**	−0.171**	−0.108**	−0.327**	0.452**
	2005	1.0000	0.558**	−0.638**	−0.391**	−0.637**	0.657**
Y_2_	2003		1.0000	−0.176	−0.280**	−0.341**	0.302**
	2004		1.0000	−0.045**	−0.047**	−0.099**	0.122**
	2005		1.0000	−0.453**	−0.294**	−0.422**	0.377**
Y_3_	2003			1.0000	0.787**	0.113	0.010
	2004			1.0000	0.071**	0.127**	−0.089**
	2005			1.0000	0.582**	0.514**	−0.332**
Y_4_	2003				1.0000	0.234*	−0.250*
	2004				1.0000	0.053**	−0.047**
	2005				1.0000	0.322**	−0.142
Y_5_	2003					1.0000	−0.219*
	2004					1.0000	−0.209**
	2005					1.0000	−0.354**

*Significant at the 0.05 probability level.

**Significant at the 0.01 probability level.

N = 105, 129 and 146 for year 2003 to 2005, respectively.

### Path analyses of Y_1_ to Y_5_ with Z

The results of the path analyses showed that all five seed yield components presented a strong, highly significant direct effect on Z in at least two of the years (Table 3). However, the direct effect of Y_1_ on Z was strong and positive (highlighted in bold) for all 3 years (2003, 2004 and 2005 were at *P* < 0.0001), where the coefficients were 0.480, 0.423 and 0.777, respectively. Therefore, Y_1_ had the largest contribution to Z. Y_5_ in 2004 (−0.065 at *P* < 0.001), Y_3_ in 2003 (0.454 at *P* < 0.05) and Y_4_ in 2003 (−0.371 at *P < *0.05) had weak but statistically significant direct effects on Z.

**Table 3:** Path analysis showing direct and indirect effect of Y_1_–Y_5_ to Z (*Pascopyrum smithii* Schreb.).

	year	Indirect effect via				
		→ Y_1_ → Z	→ Y_2_ → Z	→ Y_3_ → Z	→ Y_4_ → Z	→ Y_5_ → Z
Y_1_	2003	**0**.480***	0.0383	−0.1348	0.1740	−0.0239
	2004	**0**.423***	0.0077	0.0010	−0.0005	0.0213
	2005	**0**.777***	0.0257	−0.0517	−0.0391	−0.0554
Y_2_	2003		**0**.**088**	−0.0799	0.1039	−0.0188
	2004		**0**.061***	0.0003	−0.0002	0.0064
	2005		**0**.**046**	−0.0367	−0.0294	−0.0367
Y_3_	2003			**0**.454**	−0.2920	0.0062
	2004			**−0**.**006**	0.0004	−0.0083
	2005			**0**.**081**	0.0582	0.0447
Y_4_	2003				**−0**.371*	0.0129
	2004				**0**.**005**	−0.0034
	2005				**0**.**100**	0.0280
Y_5_	2003					**0**.**055**
	2004					**−0**.065**
	2005					**0**.**087**
Total direct effect		**1**.**68**	**0**.**195**	**0**.**529**	**−0**.**266**	**0**.**077**
Total effect		1.68	0.2667	0.2272	−0.2907	0.05

*Significant at the 0.05 probability level.

**Significant at the 0.01 probability level.

The direct effects of Y_1_–Y_5_ to Z are highlighted in bold (on the main diagonal cells); Arrows indicate the direction of the effect.

Regarding the contributions of Y_1_–Y_5_ to Z, the strongest positive indirect influence was presented by the pathway from Y_1_ via Y_4_ (the coefficient was 0.1740 in 2003), and the second indirect affect was demonstrated by Y_2_ through Y_4_ (0.1039 in 2003). In order of decreasing magnitude, Y_3_ via Y_4_ (0.0582 in 2005) and Y3 via Y5 (0.0447 in 2003) were observed. The strongest negative indirect influence on Z was Y_2_ via Y_3_ (−0.0799 in 2003), and the second indirect affect was demonstrated by Y_1_ through Y_3_ (−0.0517 in 2005). Y_3_ had a negative and marginal direct influence on yield in 2004 and positive effects during the other 2 years on seed yield (Table 3), Y_3_ had the smallest contribution to Z (Table 3). In summary, the order of contributions of the five seed yield components were Y_1_ > Y_3_ > Y_2_ > Y_5_ > Y_4_, and the total direct effects were 1.68, 0.529, 0.195, 0.077 and −0.266, respectively (Table 3). With Y_4_, the influence was negative, but the overall order of effects was Y_1_ > Y_2_ > Y_3_ > Y_5_ > Y_4_ (1.68, 0.2667, 0.2272, 0.05 and −0.2907, respectively, Table 3).

### Ridge regression models of seed yield and 5 seed yield components

**Figure 1:**
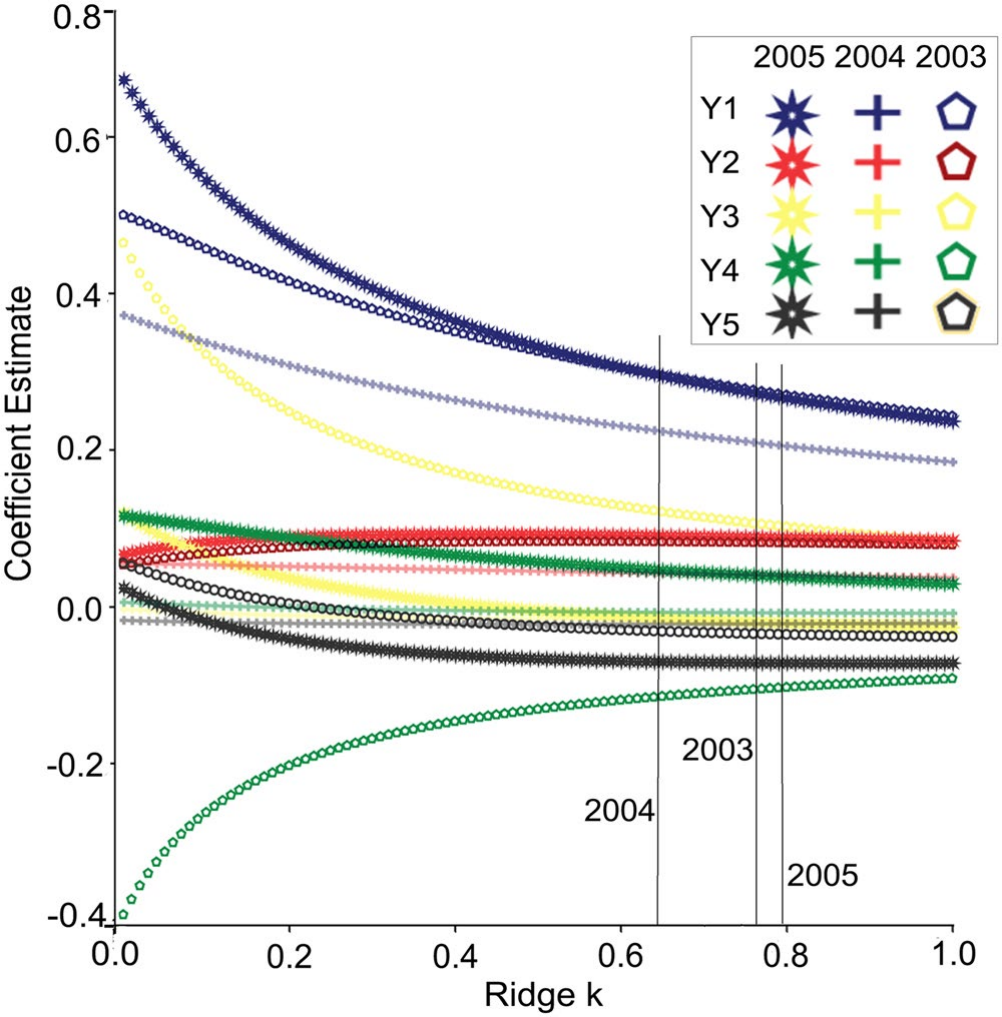
Ridge traces of standard partial regression coefficients for increasing values of k for five yield components for year 2003, 2004 and 2005, respectively. Y_1_ to Y_5_ are fertile tillers m^−2^, spikelets per fertile tillers, florets per spikelet, seed numbers per spikelet and seed weight, respectively.

Seed yield (Z) was highest in 2003 followed by 2004 and 2005 (Table 4). Y_1_ was highest in 2003 and produced the highest Z. It has been suggested that the value of the ridge parameter K^11^ should be determined using ridge traces (Fig. 1). For 2003, 2004 and 2005, the curves for Y_1_ to Y_5_ showed estimated k values at 0.77, 0.64 and 0.79, respectively (Fig. 1). The ridge regression models A, B and C for 2003, 2004 and 2005, respectively (Table 4), were as follows:

**Table 4:** Duncan’s Multiple Range Test of the *Pascopyrum smithii* seed yield (Z) and yield components (Y_1_–Y_5_) for the 3 years and of the ridge regression coefficients.

	year		N	Y_1_	Y_2_	Y_3_	Y_4_	Y_5_	Z
**Duncan’s Multiple Range Test**									
	2003		105	646.14 a	17.5805 a	12.5887 a	5.7998 a	4.8564 a	822.21 a
	2004		129	236.44 b	18.3464 c	7.3498 b	4.4944 b	4.5575 ab	492.49 b
	2005		146	401.85 c	18.7094 b	7.5531 c	4.7874 c	4.5718 b	473.47 c
	F Value			363.244	27.27	31.13	29.11	159.52	132.88
	Pr > F			<0.0001	<0.0001	<0.0001	<0.0001	<0.001	<0.0001
**Ridge regression coefficients**									
k	year	Intercept		Y_1_	Y_2_	Y_3_	Y_4_	Y_5_	Z
0.77	2003	529.067		0.371	19.944	15.127	−55.349	−29.703	−1
0.64	2004	444.094		0.358	2.075	−2.205	−0.920	−4.078	−1
0.79	2005	271.685		0.381	14.551	−7.485	18.647	−48.165	−1

Means with the same letter are not significantly different at Alpha = 0.05.

A. $${\rm{Z}}=529.067+0.371\times {Y}_{1}+19.944\times {Y}_{2}+15.127\times {Y}_{3}-55.349\times {Y}_{4}-29.703\times {Y}_{5}$$

(Ridge k = 0.77; *F* = 8.274 *Pr* < 0.0001)

B. $${\rm{Z}}=444.094+0.358\times {Y}_{1}+2.075\times {Y}_{2}-2.205\times {Y}_{3}-0.920\times {Y}_{4}-4.078\times {Y}_{5}$$

(Ridge k = 0.64; *F* = 114.768 *Pr* < 0.0001)

C. $${\rm{Z}}=271.685+0.381\times {Y}_{1}+14.551\times {Y}_{2}-7.485\times {Y}_{3}+18.647\times {Y}_{4}-48.165\times {Y}_{5}$$

(Ridge k = 0.79; *F* = 10.797 *Pr* < 0.0001)

The highest absolute values of the ridge regression coefficients for Y_4_, Y_3_ and Y_2_ occurred in 2003, and for Y_5_ and Y_1_, the highest values occurred in 2005 (Table 4). For a reliable model from the data of three successive years, the 380 samples of Z with Y_1_ to Y_5_ in the database were transformed using the natural logarithm: S = ln Z, C_1_ = ln Y_1_, C_2_ = ln Y_2_, C_3_ = ln Y_3_, C_4_ = ln Y_4_ and C_5_ = ln Y_5_.

Then, the ridge regression model was obtained with S and C_1_-C_5_ as follows: (The variance analysis and the parameter estimates are listed in Tables 5 and 6 respectively).

1 $$\begin{array}{c}{\rm{S}}=5.219+0.211\times {C}_{1}+0.095\times {C}_{2}+0.005\times {C}_{3}-0.004\times {C}_{4}-0.295\times {C}_{5}\\ ({\rm{N}}=380,F=209.514,Pr < 0.0001)\end{array}$$

Thus,

2 $$\mathrm{ln}\,{\rm{Z}}=5.219+0.211\times \,\mathrm{ln}\,{Y}_{1}+0.095\times \,\mathrm{ln}\,{Y}_{2}+0.005\times \,\mathrm{ln}\,{Y}_{3}-0.004\times \,\mathrm{ln}\,{Y}_{4}-0.295\times \,\mathrm{ln}\,{Y}_{5}$$

**Table 5:** Analysis of variance for dependent variable Z_actual_ with the 5 seed-yield components of the 380 samples.

Source	DF	Sum of squares	Mean square	F value	Pr > F
Model	1	43398208.445	43398208.445	1047.004	<0.0001
Error	379	183167057.556	41449.889		
Corrected total	380	226565266.002

**Table 6:** Parameter estimates for Z_estimated_.

Variable	DF	Parameter estimate	Standard error	t value	Pr > |t|
Intercept	1	−230.174	24.195	−9.513	<0.0001
Z_estimated_	1	1.568	0.048	32.357	<0.0001

Model (2) was transformed to an exponential function:

3 $${\rm{Z}}=181.272\times {Y}_{1}^{0.21}\times {Y}_{2}^{0.1}\times {Y}_{3}^{0.01}\times {Y}_{4}^{-0.01}\times {Y}_{5}^{-0.30}$$

Equation (3) was used to estimate the seed yield of all 380 samples, and the results were denoted as Z_estimated_. The actual seed yields were denoted as Z_actual_. To test the accuracy, the values of Z_actual_ to Z_estimated_ were used for linear regression (analyses of the variance is shown in Tables 5 and 6). The linear model was as follows:

4 $${\rm{Zactual}}=-\,230.174+1.568\times {Z}_{estimated}\,({\rm{N}}=380,\,{\rm{F}}=1047.004,\,{\rm{\Pr }} < 0.0001)$$

Then, via formula (4), the model was adjusted

5 $${\rm{Zactual}}=-\,230.174+284.235\times {Y}_{1}^{0.21}\times {Y}_{2}^{0.1}\times {Y}_{3}^{0.01}\times {Y}_{4}^{-0.01}\times {Y}_{5}^{-0.30}$$

**Figure 2:**
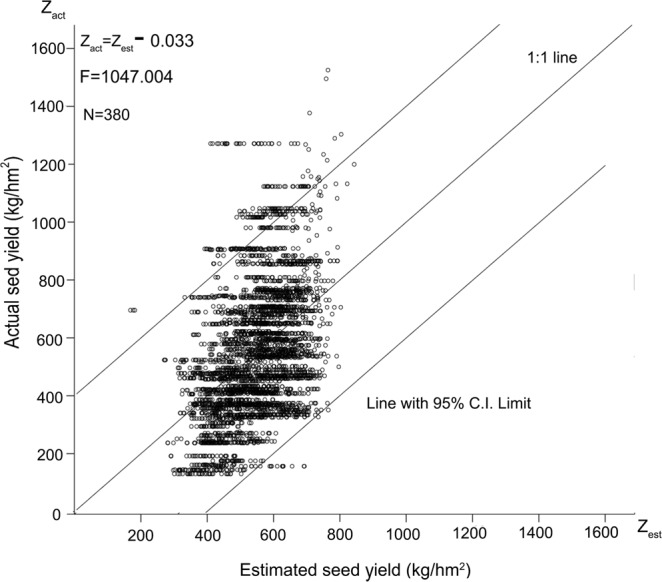
Scatter plot to fit regression line of actual and estimated seed yield adjusted by Zactual. = −230.174 + 284.235 × Y_1_^0.21^ × Y_2_^0.1^ × Y_3_^0.01^ × Y_4_^−0.01^ × Y_5_^−0.30^ of the 3 years. It is superimposed on the 1:1 line.

The variance test estimated that the intercept and Z were −0.033 and 1.000, respectively (Table 7), which are presented in Fig. 2, superimposed on the 1:1 line.

**Table 7:** Parameter estimates for Z_estimated_ after adjustment by linear regression.

Variable	DF	Parameter estimate	Standard error	t value	Pr > |t|
Intercept	1	−0.033	17.164	−0.002	0.998
Z_estimated_	1	1.000	0.031	32.357	<0.0001

**Figure 3:**
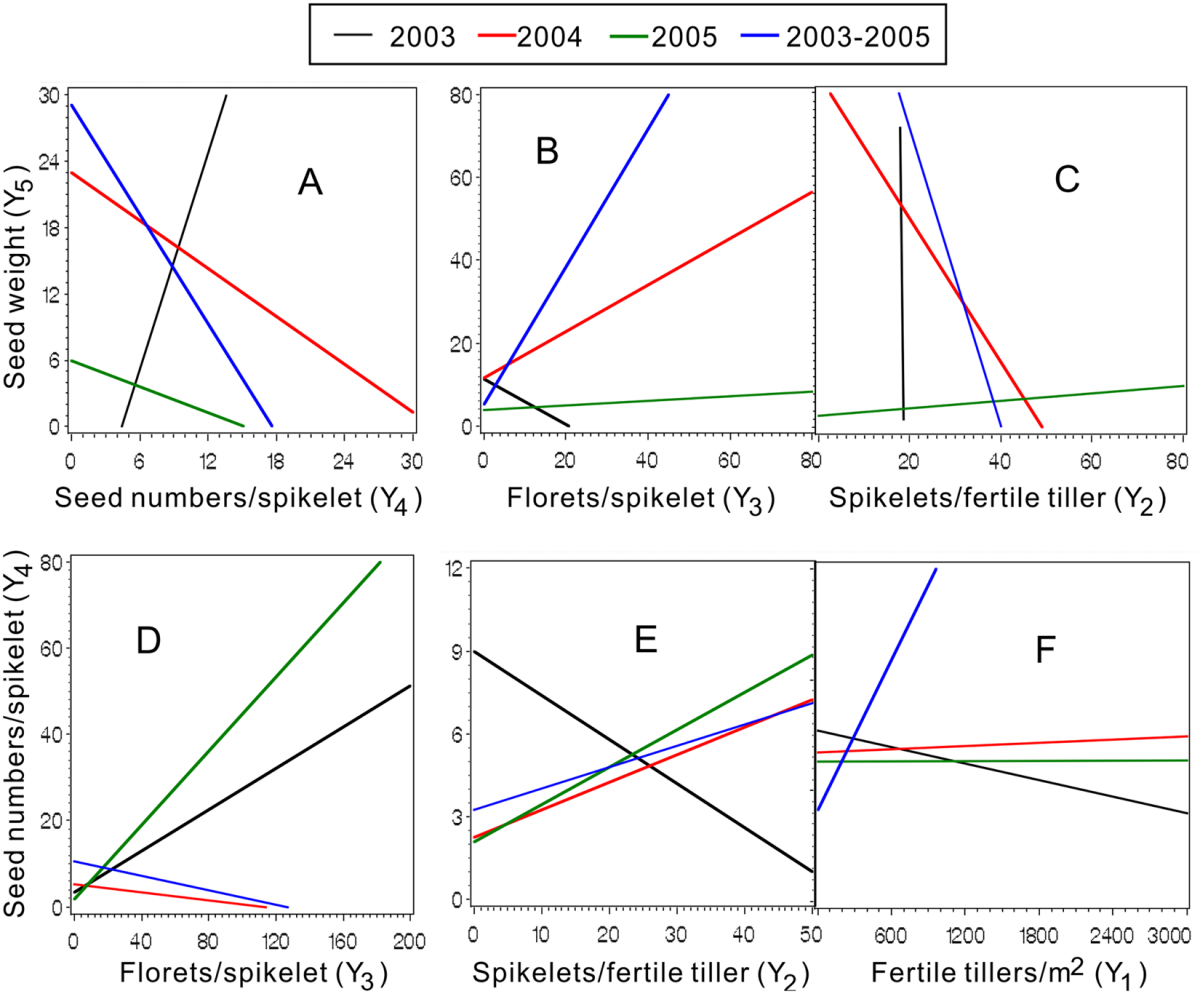
Ridgelines of the response surface models showed the synergism and antagonism through Y_4_ (**A**) Y_3_ (**B**) and Y_2_ (**C**) to Y_5_ and Y_3_ (**D**) Y_2_ (**E**) and Y_1_ (**F**) to Y_4_.

**Figure 4:**
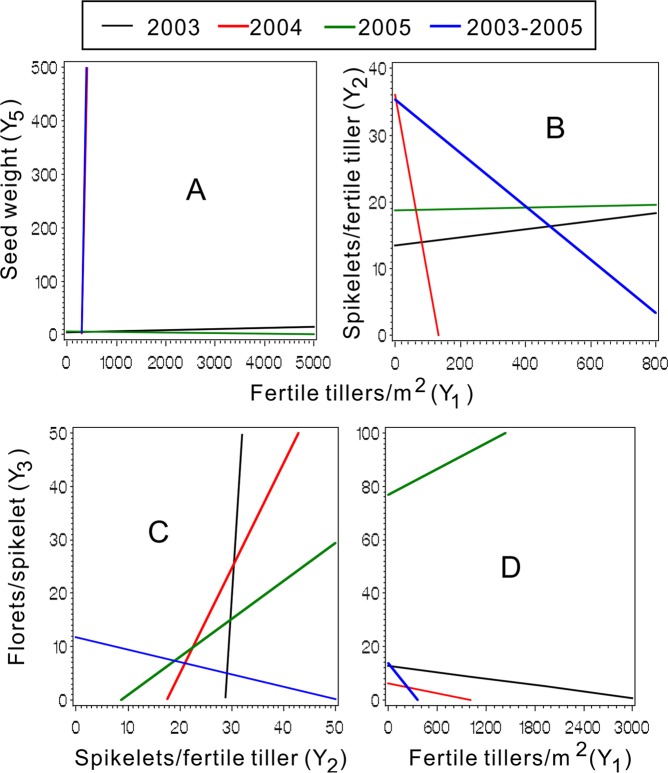
Ridgelines of the response surface models showed the synergism and antagonism through Y_1_ to Y_5_ (**A**) and Y_2_ (**B**), Y_2_ (**C**) and Y_1_ (**D**) to Y_3_.

We determined the antagonistic and synergistic effects among the Ys on Z using pairwise models (Figs. 3 and 4). In addition, the results of synergism and antagonism among the Y_1_ to Y_5_ on Z are discussed (Figs. 3 and 4).

## Discussion

In this study, precipitation, temperature, and sunlight during the crop-growing period from March to early September for the three years of the study were provided by the Jiuquan Meteorological Observatory of Gansu Province, China (Fig. S1). Temperature and precipitation during the crop growing seasons from 2003–2005 were near the average values for the past ten years in this location (Table S14). Thus, this data should accurately represent the natural field conditions.

The average *Pascopyrum smithii* seed yield (Z) and the yield components (Y_1_, Y_2_, Y_3_, Y_4_ and Y_5_) were very different from 2003 to 2005 (Table 8), mainly owing to climatic conditions (Supplementary Fig. 1), e.g., large precipitation differences between 2003, 2004 and 2005. Moreover, higher rainfall in June, which occurred during the seed growth period, was partly responsible for higher seed yields because it favored pollination and grain filling. As another example, the highest recorded rainfall was in March 2005 (28.2 mm), together with a low air temperature. These factors promoted vegetative growth and significantly decreased Y_1_ (Table 4), which consequently resulted in the lowest values for Z. The highest values for Z appeared in 2003 because the mild climate and adequate water supply were conducive to crop tillering and yield improvement. In comparison, the higher Z in 2004 corresponded to higher temperatures and adequate water in June and July in 2004 compared to 2005. However, Y_1_ and Y_3_ weakly decreased along with age from 2003 to 2005, which may have been due to genetic factors, while larger difference indicate the role of environment^30,31^. Fertile tillers m^−2^ (Y_1_), florets/spikelet (Y_3_), and seed numbers/spikelet (Y_4_) were higher in 2003 compared to 2004 and 2005, whereas spikelets/fertile tillers (Y_2_) was lower in 2003 than in 2004 and 2005. Seed weight (Y_5_), in contrast, was similar across the years. For each of the traits evaluated, descriptive statistics, including the coefficient of variation (CV), mean, minimum, maximum, Std-Dev and Std-Error values are summarized in Table 8. These indicators have strong differences for improving the expression of traits and provide a good opportunity to cultivate excellent species. The variabilities in seed yield and the yield components in 2003, 2004 and 2005 may have been caused by a stand age divergence, air temperature differences, interactions between soil fertility and climatic conditions, or combinations thereof.

**Table 8:** Statistics of Y_1_~Y_5_, Z (*Pascopyrum smithii* Schreb.) for years 2003~2005.

Variable	year	N	Mean	Std-Dev	Std-Error	Minimum	Maximum	CV	Pr > |t|
Y_1_	2003	105	674.4784	178.2814	17.3985	192.38	1153.33	0.27	<0.0001
	2004	129	267.7532	126.9550	1.9660	34	916	0.48	<0.0001
	2005	146	434.7001	161.2333	13.3438	167.17	830.67	0.37	<0.0001
Y_2_	2003	105	17.6083	0.9908	0.0967	15.21	20.21	0.06	<0.0001
	2004	129	18.8091	4.3803	0.0678	9	40	0.23	<0.0001
	2005	146	18.7603	1.3885	0.1149	15.63	22.03	0.08	<0.0001
Y_3_	2003	105	12.6991	1.6743	0.1634	8.84	17.12	0.13	<0.0001
	2004	129	7.5309	1.6297	0.0252	3	16	0.22	<0.0001
	2005	146	7.5742	0.5718	0.0473	6.4	9.03	0.08	<0.0001
Y_4_	2003	105	5.8180	0.4572	0.0446	4.39	6.64	0.08	<0.0001
	2004	129	4.8165	1.6596	0.0257	1	13	0.35	<0.0001
	2005	146	4.8125	0.4960	0.0410	3.7	6.33	0.10	<0.0001
Y_5_	2003	105	4.8644	0.2823	0.0275	4.336	5.802	0.06	<0.0001
	2004	129	4.5932	1.1330	0.0175	3.19	44.61	0.25	<0.0001
	2005	146	4.5838	0.3326	0.0275	3.91	5.27	0.07	<0.0001
Z	2003	105	856.1210	241.0244	23.5216	371.69	1525.07	0.28	<0.0001
	2004	129	539.4421	220.5409	3.4152	130.15	1271.088	0.41	<0.0001
	2005	146	523.4232	225.8983	18.6955	130.15	1271.088	0.43	<0.0001

Correlation analysis considers a mutual association with no regard to causation, whereas path analysis specifies causes and measures their relative importance^32^. Because the total correlations between the predictor variables and the response variable, which are partitioned into direct and indirect affects, and the direct and indirect effects of the yield factors can be determined through path analysis, and knowledge on the direct and indirect correlations, especially of the yield, allows breeders to use this additional information to discard or promote genotypes of interest^33^. Alvi *et al*.^34^ and Asghari-zakaria *et al*.^35^ used path analysis to enable the identification of traits that are useful for an evaluation standard in increasing crop yield. Cruz *et al*.^36^ defines the path coefficient, or cause and effect analysis, as a standardized regression coefficient^37^, because path analysis is composed of an expansion of multiple regressions when complex interrelationships are involved. Thus, path analysis has been found to be a useful technique of statistical analysis specially designed to quantify direct and indirect trait association with yield^38^. In this study, path analysis indicates that the total direct effects of Y_1_, Y_2_, Y_3_ and Y_5_ have highly significant positive correlations with Z, but Z is negatively correlated with Y_4_ (Table 3). The explanation for why there is a weak negative correlation between the seed number (Y_4_) and seed yield (Z) might matter to a high density cultivation and soil nutrient limitation^7^. This finding is consistent with the literature, e.g., the yield of mechanically harvested rapeseed (*Brassica napus* L.) can be increased by optimum plant density and row spacing^39^. Table 3 shows that Y_1_ and Y_2_ have the largest correlation coefficient and contribution rate, which is consistent with the natural law of plant growth and biological theory. Nevertheless, the correlation of Z with Y_3_ (Table 1), which was not significant, is probably due to the effects of aging and climate during the individual years and to the field management systems that were repeated yearly^40^. However, Y_3_ did not significantly contribute to Z partly because Y_3_ was mostly under genetic control^40^. This discovery implies that Y_3_ is the sub group that should be taken into account if high quality forage is the target of the breeding system. Nevertheless, Y_1_ was the most critical and available group that significantly contributed to Z (*P* < 0.001): the coefficients were 0.480, 0.423, and 0.777 for 2003, 2004 and 2005, respectively. This finding is consistent with previous studies in fescues^7,41^, Russian wild rye^42,43^, Zoysia grass^1^, gramineous plants^5^, and white clover^40,44,45^. Additionally, path analysis uncovered relationships between the components and the yield that are consistent with previously reported results^45^. The interrelationships among Y_1_ to Y_5_ indicated that Y_4_ and Y_5_ had remarkable negative relationships with seed yield (Z; r = −0.03* and r = −0.192**). Y_1_ and Y_2,_ as indicated above, had significant and positive correlations with Z. Because of the existence of positive and negative correlations between the seed yield components, simple linear relationships between two components by a correlation analysis cannot be successfully predicted by the measurements. However, with standardized variables, a path analysis can determine the relative importance of direct and indirect effects on seed yield. The results of this study further emphasize that as the plants aged during the successive experimental years, Y_1_, Y_2_ and Y_3_ decreased significantly, whereas Y_4_ and Y_5_ increased. This finding is consistent with the results of previous research^40^. This result also implies that Y_4_ and Y_5_ should and could be effectively improved if the values of Y_1_, Y_2_ and Y_3_ are lower than normal.

Ridge regression and multiple-regression analyses were applied to avoid high inter-correlation and multico-linearity among the variables^46–48^. The significant correlation coefficients (*P* < 0.001 and 0.01), path analyses, and ridge regressions of the multifactor orthogonal experimental design and large sample statistical analysis in the field experiments show that the models are reliable^48^. In addition, ridge regression effectively overcomes the problem of highly multi-correlated predictor variables (seed yield components)^48,49^. This method is the most effective and practical for the current field scientific research^43^. Unfortunately, owing to the aging of the plants, designed field management and climate conditions, the coefficients of the ridge regression models in individual years are variable, ranging from −55.349 to 19.944 (Table 4). An original exponential model was found to estimate Z via Y_1_ through Y_5_. First, the final algorithm model [exponential Eq. (4)] was deduced using data for 380 plots from various growth regimes in the three successive years. Moreover, all three ridge regression models [The ridge regression models A, B and C] for the individual years were significant (*P* < 0.001), and they all had coefficients matching the contributions of the five Ys to Z. This result is explained through the relationship between the path analysis and ridge regression analysis, and the test methods and results are more credible. In addition, the contributions in absolute value of the five seed yield components to the seed yield are in the following order: Y_1_ > Y_3_ > Y_4_ > Y_2_ > Y_5_ (1.68, 0.529, −0.266, 0.195 and 0.077, Table 3). The total direct effects with Y_4_ having negative results, showed that the total influence order, in absolute values, is Y_1_ > Y_4_ > Y_2_ > Y_3_ > Y_5_ (1.68, −0.2907, 0.2667, 0.2272 and 0.05, Table 3). However, the two pairs of results are from the same database. The ridge analysis values analytically combine the effects of all Ys, especially the effects of aging and climate, to address the variation in Z for the three years, whereas the path analysis includes separate analytic effects of the individual three years.

The antagonistic and synergistic effects between Ys on Z were investigated using pairwise quadratic regression models (Table 9, Figs. 3 and 4). The lines for 2004 (red) and 2003–05 (blue) showed uniform orientations, except for Y_4_ & Y_2,_ among the ten relation schema subgraphs (Figs. 3 and 4), as did the lines for 2005 (green), except for Y_5_ & Y_4_. The subgraphs indicated that Y_4_ & Y_5_ (Fig. 3A), Y_2_ & Y_5_ (Fig. 3C), Y_3_ & Y_4_ (Fig. 3D), Y_1_ & Y_5_ (Fig. 4B) and Y_1_ & Y_3_ (Fig. 4D) had antagonistic effects on Z, as the ridgelines were at k < 0 (Table S4)^49^. Conversely, the red and blue lines that also had the same directions indicated that Y_3_ & Y_5_, Y_2_ & Y_4_ and Y_1_ &Y_4_ had synergetic effects on Z (Fig. 3B,E,F) at k > 0 in the ridgelines. The more Y_2_, less Y_5_ dynamic (Fig. 3C), which was also evident for Y_3_ & Y_4_ (Fig. 3D), were mostly caused by feedforward compensation at the biological level and by the soil nutrient limitation. In Fig. 4A, the blue and red lines almost overlap and are nearly perpendicular to the horizontal axis, whereas the green and black (2003) lines are also partly overlapped and nearly perpendicular to the longitudinal axis. This may be due to genetic constraints on Y_5_ in a certain range of growth. In this range, Y_1_ has the optimal value. The increase in Y_1_-derived Y_2_ and the Y_3_ decrease (Fig. 4B,D) were consistent with the soil nutrient limitation. For 2003, 2004, 2005 and 2003–2005, the interactions between Y_2_ & Y_3_ gradually changed from synergetic to antagonistic (Fig. 4C). As important seed yield traits of the plant, Y_2_ & Y_3_ are regulated by genetics. Further investigations will be needed to verify that these changes are probably due to aging of the grass.

**Table 9:** Coefficients of pair wised models among the seed yields components.

		Y_a_^2^	Y_b_^2^	Y_a_*Y_b_	Y_a_	Y_b_	Constant	*F* Value	*P* > F
2003	Y_1_Y_2_	0.001	23.052	−0.276	4.505	−622.169	4490.5	10.30	<0.0001
N = 146	Y_1_Y_3_	0.0003	4.719	0.035	−0.054	−119.522	1182.2	10.63	<0.0001
	Y_1_Y_4_	0.0003	139.517	0.157	−0.597	−1719.56	5747.4	10.09	<0.0001
	Y_1_Y_5_	0.001	−120.43	0.387	−2.25	960.74	1120.8	9.91	<0.0001
	Y_2_Y_3_	−1.795	0.778	1.913	112.84	−45.34	−551.4	1.96	**0**.**092**
	Y_2_Y_4_	−4.117	164.24	36.83	−9.73	−2642.62	8321.6	3.61	0.0048
	Y_2_Y_5_	22.54	95.228	189.01	−1667.9	−4346.47	25926.8	3.16	0.0110
	Y_3_Y_4_	26.74	446.78	−210.7	633.1	−2887.0	5704.3	6.09	<0.0001
	Y_3_Y_5_	1.35	−164.8	−115.7	526.52	2969.8	−9435.1	2.37	0.0455
	Y_4_Y_5_	170.1	−79.72	−381.8	−232.4	2935.4	−5155.2	4.05	0.0022
2004	Y_1_Y_2_	−0.001	−0.322	−0.14	1.545	19.74	76.6	177.85	<0.0001
N = 4221	Y_1_Y_3_	−0.001	1.280	0.012	1.268	−14.19	345.5	173.16	<0.0001
	Y_1_Y_4_	−0.001	−2.985	−0.001	1.332	33.41	236.4	172.92	<0.0001
	Y_1_Y_5_	−0.001	0.145	0.388	−0.583	−114.70	863.0	187.95	<0.0001
	Y_2_Y_3_	−0.545	−0.064	0.864	20.767	−13.58	363.1	15.89	<0.0001
	Y_2_Y_4_	−0.531	−3.36	−0.435	28.73	42.79	145.0	13.14	<0.0001
	Y_2_Y_5_	−0.45	2.228	2.483	11.153	−155.42	969.0	69.62	<0.0001
	Y_3_Y_4_	0.063	−3.082	−0.153	0.997	32.46	486.3	6.54	<0.0001
	Y_3_Y_5_	0.580	2.554	−3.852	12.41	−91.55	931.4	64.87	<0.0001
	Y_4_Y_5_	−3.292	2.408	−1.797	42.35	−109.45	932.2	64.15	<0.0001
2005	Y_1_Y_2_	0.001	−20.61	0.049	−0.093	773.29	−6964.8	16.11	<0.0001
N = 105	Y_1_Y_3_	−0.001	−1.135	0.035	1.456	178.76	−1293.1	19.95	<0.0001
	Y_1_Y_4_	−0.001	−148.57	0.003	1.562	1498.01	−3674.6	18.84	<0.0001
	Y_1_Y_5_	−0.001	−312.09	−0.776	5.363	3265.24	−8375.3	15.32	<0.0001
	Y_2_Y_3_	−18.17	−33.81	39.38	448.06	−205.19	−3504.8	8.40	<0.0001
	Y_2_Y_4_	−25.70	−185.18	−1.496	1024.87	1797.63	−13779.0	5.56	<0.0001
	Y_2_Y_5_	−22.65	−238.08	−3.591	911.35	2198.11	−13273.1	7.56	<0.0001
	Y_3_Y_4_	−82.24	−148.47	−64.47	1500.05	1885.23	−9322.8	4.18	0.0014
	Y_3_Y_5_	−41.82	−326.69	−51.10	900.02	3212.32	−9896.0	6.52	<0.0001
	Y_4_Y_5_	−82.85	−327.26	−303.08	2198.81	4280.04	−14104.2	6.00	<0.0001
2003–05	Y_1_Y_2_	0.0005	−0.301	−0.023	1.073	21.52	109.6	214.62	<0.0001
N = 4426	Y_1_Y_3_	−0.001	0.823	0.059	0.485	−21.42	479.1	227.53	<0.0001
	Y_1_Y_4_	0.0004	−2.454	0.045	0.490	16.60	374.4	212.55	<0.0001
	Y_1_Y_5_	−0.0003	0.224	0.557	−1.886	−166.30	1161.5	237.93	<0.0001
	Y_2_Y_3_	−0.648	4.445	0.602	27.02	−69.91	483.1	26.47	<0.0001
	Y_2_Y_4_	−0.661	−4.380	−0.699	35.11	61.48	42.3	13.73	<0.0001
	Y_2_Y_5_	−0.613	1.581	2.194	19.10	−118.23	759.8	57.30	<0.0001
	Y_3_Y_4_	4.169	−3.831	1.110	60.67	31.19	691.9	16.31	<0.0001
	Y_3_Y_5_	4.995	2.425	−2.908	−51.60	−92.83	1133.8	73.72	<0.0001
	Y_4_Y_5_	−4.448	1.841	−1.059	54.13	−83.82	795.4	51.98	<0.0001

## Conclusions

Algorithmic models were developed to describe the seed yield and yield components needed to improve the seed yield of *Pascopyrum smithii*. Significant positive correlations were observed between seed yield (Z) and spikelets/fertile tillers (Y_2_) and fertile tillers m^−2^ (Y_1_), and negative correlations were found with seed number/spikelet (Y_4_). The inter-correlation among these components were significant in 2004 and 2005.

The model of seed yield with its 5 components, based on a large sample size from an orthogonal experimental design in *Pascopyrum smithii*, was:

$${\rm{Z}}=-\,230.174+284.235\times {Y}_{1}^{0.21}\times {Y}_{2}^{0.1}\times {Y}_{3}^{0.01}\times {Y}_{4}^{-0.01}\times {Y}_{5}^{-0.30}$$

This model can be used to accurately estimate the seed yield with five yield components.

The total direct effects of fertile tillers, florets/spikelet, spikelets/fertile tiller and seed weight on seed yield were positive, and fertile tillers was the largest contributor. The contributions in decreasing order were fertile tillers(Y_1_) > seed number/spikelet (Y4) > spikelets/fertile tiller (Y_2_) > florets/spikelet (Y_3_) > seed weight(Y_5_). Fertile tillers (Y_1_) was one of the most important factors that played a key role in seed production. Therefore, selection for high seed yield through direct selection for large fertile tillers (Y_1_), florets/spikelet (Y_3_)_,_ and seed number/spikelet (Y4) would be effective and reliable for breeding, based on the experimental data of three years of continuous field experiments. Finally, this research laid the foundation for the basic theory of restoration ecology in arid regions and for the promotion of plant carbon cycles to reduce the greenhouse effect. Further studies should be focused on changes in seed yield based on different climatic conditions and site locations.

## Materials and Methods

### Experimental site description

The cultivar “Rosana” of *P*. *smithii* which is commonly planted was introduced from the United States in 2002[the 948 project (202009) of the Ministry of Agriculture of China]. Field experiments were conducted over three years (2003–2005) at the China Agricultural University Grassland Research Station located at the Hexi Corridor, in Jiuquan, Gansu province, northwestern China (39°37′ N latitude and 98°30′ E longitude; altitude 1480 m). Soil at the site is classified as a Mot-Cal-Orthic Aridisol in the Chinese system and as a Xeric Haplocalcid in the USDA soil classification system^50^. The plots used in this experiment were planted with alfalfa (*Medicago sativa* L.) in the preceding season. The 6000 m^2^ experimental site was tilled using a chisel plow in the fall and disk-harrowed in the spring for seedbed preparation. *Pascopyrum smithii* seeds were planted on April 23, 2002 at a depth of 2.5 cm. The seeding rate was 500 seeds m^−2^, and the space between rows was 0.45 m. Initial fertilizer was applied in a band that was 6 cm deep and 5 cm to the side of the seed furrows at a rate of 104 kg ha^−1^ N and 63 kg ha^−1^ P_2_O_5_. There was no seed yield in autumn 2002. Initial chemical characteristics of the soil (0–20 cm) were: pH = 8.39; NH_4_^+^ = 32.32 mg kg^−1^; NO_3_^−^ = 20.09 mg kg^−1^; alkali hydrolysable nitrogen = 118.30 mg kg^−1^; available phosphorus = 36.56 mg kg^−1^; available potassium = 130.30 mg kg^−1^; total nitrogen = 0.764 g kg^−1^; total phosphorus = 0.814 g kg^−1^; total potassium = 12.52 mg kg^−1^; organic matter = 10.32 g kg^−1^ (Table S1).

### Experimental design

The Orthogonal Experimental Design (OED) method is typically used to study the comparative effectiveness of multiple intervention components simultaneously; OED with both Orthogonal Array (OA) and Factor Analysis (FA) makes it possible to discover the optimum combinations with only several tests^51,52^. Thus, to simulate various growing conditions, we used six groups (A to E) (Table S2). Because OED is characterized by equilibrium dispersion, we were able to design experiments to find the best combination of treatments with a minimum of tests. Additionally, OED allowed us to transform complex multi-factor data to single factor analysis. We used a multi-factorial orthogonal design for field plots based on the six groups (Table S2)^53^, giving a total of 380 experimental plots (each with an area of 28 m^2^), under various field management treatments (Table S2) within a total field area of 4100 m^2^. Plots were irrigated five times during the growing season at the following growth stages: vegetative phase jointing, stem formation, ear formation and flowering, respectively. Fertilization was carried out before sowing and again before spring regrowth the following year.

### Data collection

Ten samples along 1 m of each row were randomly selected to measure the five seed yield components from anthesis to seed harvest from 2003 to 2005. Plants that were 1 m or less from the edge of the plot we not sampled. Seed yield components and seed yield data for each plot were collected as follows: fertile tillers m^−2^ (Y_1_) were measured from ten randomly selected 1-m row samples, and 30 to 36 fertile tillers and 27 to 54 spikelets were randomly selected for measuring spikelets/fertile tillers (Y_2_), florets/spikelet (Y_3_) and seed numbers/spikelet (Y_4_). When the seed heads were ripe, 4 samples from 1 m of the row length were separately threshed by hand, the yield of clean seed for each sample was weighed, and the seed moisture content was confirmed as 7 to 10% for converting into seed yield (kg hm^−2^) (Z). Ten lots of 100 seeds each were collected to determine mg seed weight (Y_5_). The total number of samples (n) used to measure Y_1_ to Y_5_ and Z were 3800, 13605, 11085, 10770, 3800, and 1520, across all 3 years (Table S3). The sample size for individual years is shown in Supplementary Table 3, and the experimental databases were established with Visio FoxPro (Version 6.0).

### Statistics and analytical methods

Path coefficient analysis helps to determine the direct effect of traits and their indirect contributions via other characters^38,54^. Correlation and path analysis were performed to determine the relationship among the yield and yield contributing characters. Thus, separate and combined analyses for the three years provided useful information^48^.In addition, ridge regression is a useful parameter estimation method for addressing the collinearity problem frequently arising in multiple linear regression. Ridge regression provides a means of addressing the problem of collinearity without removing variables from the original set of independent variables. Ridge regression analysis^55^ and Duncan’s multiple range tests for seed yield (Z) and yield components (Y_1_–Y_5_) were performed. The data were transformed using logarithmic and power transformations to avoid the effects of highly inter-correlated data, which would lead to multico-linearity between Y_1_–Y_5_ and Z. To establish a reliable model, all of the Z and Y_1_–Y_5_ data in Visio FoxPro, representing a total of 380 samples (plots) in the three years (i.e. 105 + 129 + 146), were taken as a natural logarithm because mathematically they did not influence the essential relationships between the variables. Analyses of variance and Pearson correlation analyses were performed using SPSS Version 19.0.

If S = InZ and C_i_ = InY_i_ (i = 1 to 5), then S and C_1_ to C_5_ were used for the ridge regression analyses, and the ridge regression model was:

6 $${\rm{S}}={\rm{C}}\times {\rm{\beta }}+{\rm{u}}$$

where S is the n × 1 vector of observations of a response variable, C is the n × p matrix of observations on p explanatory variables, β is the p × 1 vector of regression coefficients, and u is the n × 1 vector of residuals satisfying E(ū) = C’, E(uu’) = δ^2^I.

It is assumed that C and S have been scaled such that C’ C and S’ S are matrices of correlation coefficients. In this equation, n = 380, and p = 5. Thus,

7 $${\rm{LnZ}}=(\mathop{\sum }\limits_{i=1}^{5}\,\mathrm{Ln}\,{Y}_{i})\times \beta +u$$

The above logarithmic model (7) was transformed to the following exponential function:

8 $${\rm{Z}}={e}^{a}\times \mathop{\prod }\limits_{i=1}^{5}({Y}_{i}^{\beta }),$$

where α and β are constants.

Formula (8) was used to estimate the Z of all 380 samples, which is denoted as Z_estimated_; the actual seed yield is denoted as Z_actual_.

A general linear regression model was used to assess the Z_actual_ compared to the Z_estimated_, and an analysis of variance was used to assess the dependent variable Z_actual_ and the parameter estimates of Z_estimated_. The linear regression model is:

9 $${{\rm{Z}}}_{{actual}}={\rm{\beta }}+{\rm{K}}\times {Z}_{estimated}$$

So, via formula (9), the model was adjusted to:

10 $${\rm{Z}}={\rm{\beta }}+{\rm{k}}\times {e}^{a}\times \mathop{\prod }\limits_{i=1}^{5}({Y}_{i}^{\beta }).$$

In addition, the ridge trace and appropriate scatter plots were graphed. The analyses and graphical procedures specified above were all performed using SAS Version 8.2 (Inc. 1988).

Quadratic two-variable regression models between Z and Y_1_ to Y_5_ were used as follows:

11 $${\rm{Z}}=\mathop{\sum }\limits_{i=1}^{2}({\beta }_{i\times j+1}{Y}_{j}^{i})+u(i=1,2;j=1,2),$$

Where β is a constant.

The equivalent effects of Yi and Yj were determined using:

12 $$\frac{\partial Z}{\partial {Y}_{i}}=\frac{\partial Z}{\partial {Y}_{j}}$$

which produced

13 $$\,{Y}_{j}={\rm{k}}\times {Y}_{i}\pm b,$$

where b is a constant. The presented ridgelines (13) (Figs. 3 and 4) correspond to the response surface models (11) to show the synergetic and antagonistic effects. The analyses and graphics were all performed using the SAS (v8.2) software^49^.

## Supplementary information

Supplementary Information
