# Supplementary material for: Seed yield can be explained by altered yield components in field-grown western wheatgrass (Pascopyrum smithii Rydb.)
Source: Sci Rep. 2019 Nov 29;9:17976. doi: 10.1038/s41598-019-54586-0 (PMC6884509; doi:10.1038/s41598-019-54586-0)
Supplement: Supplementary file 1 — Supplementary Information [file 41598_2019_54586_MOESM1_ESM.docx]

**Supplementary Information**

Seed yield can be explained by altered yield components in field-grown western wheatgrass (*Pascopyrum smithii Rydb.*)

**Zhao Chen**^1^, Junpeng Niu^1^, Xinlong Cao^1^, Wenbo Jiang^1^, Jian Cui^2^, Quanzhen Wang^^[[1]](#footnote-1)^^*, Quan Zhang^3^

^1^College of Grassland Agriculture, Northwest A&F University, Yangling 712100, Shaanxi Province, China.

^2^College of Life Science, Northwest A&F University, Yangling 712100, Shaanxi Province, China.

^3^Jiuquan Daye Seed Industry Co. Ltd., Jiefang Road, #325, Suzhou Qu, 735000, Jiuquan, Gansu Province, China.

To whom correspondence should be addressed:

Dr. Wang Quanzhen, College of Grassland Agriculture, Northwest A&F University (<http://www.nwsuaf.edu.cn/>), Yangling, Shaanxi Province, P. R. China

Phone: +86 (0)29-8709-1953, Fax: +86 (0)29 8709-2164, Mobile: +86 137-5994-2845, E-mail: [wangquanzhen191@163.com](mailto:wangquanzhen191@163.com)

Supplementary legends

**Supplementary Figure S1.** Monthly rainfall and average air temperature from March to August for 2003, 2004, and 2005 at the research location, in Jiuquan, Gansu province, China. **Supplementary Table S1.** The basic nutrient of experimental soil of *Pascopyrum smithii* Schreb.

**Supplementary Table S2.** Field experimental design and factors in *Pascopyrum smithii*.

**Supplementary Table S3.** The sample size of Y1 to Y5, Z for each field experimental plot from combination of the six groups experiments on *Pascopyrum smithii* Schreb.

**Supplementary Table S4.** Coding value of X_1_to X_5_ and its corresponding usage combining the six experimental designs.

**Supplementary Table S5.** Compound Matrix of L_8_（4×2^4^）Orthogonal Design. **Supplementary Supplementary Table S6.** A. 2-D optimum design (1) (Nitrogen and Phosphorus). **Supplementary Table S7.** B. 2-D-optimum design (2) (Nitrogen and Phosphorus). Six blocks total, each with a 28 m^2^ area.

**Supplementary Table S8.** C. Unique-factor orthogonal design.

**Supplementary Table S9.** Compounding matrix of unique-factor orthogonal design.

**Supplementary Table S10.** D. Bin-factor orthogonal contract blocks.

**Supplementary Table S11.** Compound matrix of Bin-factor orthogonal contract design

**Supplementary Table S12.** E. Tri-factor orthogonal rotary design.

**Supplementary Table S13.** F. Unique-factor orthogonal design [L_8_(4^1^×2^4^)].

**Supplementary Table S14.** Monthly precipitation (mm) and average temperature (°C) of the China Agricultural University Grassland Research Station located in the Hexi Corridor, Jiuquan, Gansu Province from 2003 to 2005.

**
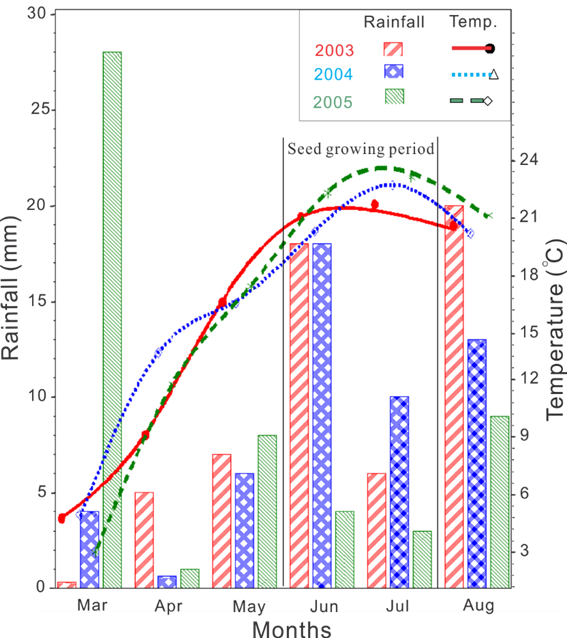
**

**Supplementary Figure S1.** Monthly rainfall and average air temperature from March to August for 2003, 2004, and 2005 at the research location, in Jiuquan, Gansu province, China. The meteorological working station in Jiuquan, Gansu province, China, provided this data.

| Depth | PH | | O.C. | T.S. | NH_4_^+^ | NO_3_^-^ | A.N. | A.P. | A.K. | T.N. | T.P. | T.K. |
| --- | --- | --- | --- | --- | --- | --- | --- | --- | --- | --- | --- | --- |
| cm | |  | g/kg | g/kg | mg/kg | mg/kg | mg/kg | mg/kg | mg/kg | g/kg | g/kg | g/kg |
| 0-20 | | 8.39 | 10.32 | 4.88 | 32.32 | 20.09 | 118.30 | 36.56 | 130.30 | 0.764 | 0.814 | 12.52 |
| 20-40 | | 8.30 | 10.33 | 7.69 | 31.09 | 12.33 | 90.88 | 18.24 | 127.00 | 0.744 | 0.733 | 11.82 |
| 40-60 | | 8.41 | 7.23 | 7.50 | 37.49 | 10.26 | 80.99 | 16.42 | 148.40 | 0.441 | 0.700 | 13.51 |

**Supplementary Table S1.** The basic nutrient of experimental soil of *Pascopyrum smithii* Schreb.

Depth: Soil Depth, O.C. : Organic matter, T.S.: Total soli, NH4^+^ : Ammonium nitrogen, NO_3_^-^: Nitrate nitrogen, A.N.: Alkali hydrolysable nitrogen, A.P.: Available phosphorus, A.K.: Available potassium, T.N.: Total nitrogen, T.P.: Total phosphorus, T.K.: Total potassium.

| Field experimental design groups | Matrices applied | Experimental factors | Repeat | plots | Matrices | Name |
| --- | --- | --- | --- | --- | --- | --- |
| A. 2-D-optimum design (1)^a^ | 2-D-optimum matrix | 2 (X_3,_ X_4_) | 3 | 18 | **Table S6** | **D16** |
| B. 2-D-optimum design (2)^a^ | 2-D-optimum matrix | 2 (X_3,_ X_4_) | 1 | 6 | **Table S7** | **D6** |
| C. Unique-factor orthogonal design | Compound matrix | 5 (X_1_-X_5_) | 1 | 36 | **Table S8, S9** | **Ww36** |
| D. Bin-factor orthogonal contract plots | Compound matrix | 2 (X_2_, X_3_+X_4_) | 1 | 22 | **Table S10, S11** | **Ew22** |
| E. Tri-factor orthogonal rotary design | Compounding matrix | 3 (X_1_, X_3_, X_6_) | 1 | 23 | **Table S12, S14** | **Se23** |
| F. Unique-factor orthogonal design | L_8_ (4^1^×2^4^) | 5 (X_1_, X_7_-X_10_) | 4 | 32 | **Table S13, S5** | **A32** |
| Control |  | -- | -- | 6-9 |  |  |
| Total |  | 10 (X_1_-X_6_) | -- | 143 |  |  |

**Supplementary Table S2.** Field experimental design and factors in *Pascopyrum smithii*. ^a^Applied N and P_2_O_5_ differently between design (1) and (2); In order, X_1_-X_10_ stand for: time of fertilizing, quantity of irrigation, applied nitrogen, applied P_2_O_5_, planted density, amount of spray plant regulator Paclobutrazol (PP333), irrigation time, density manipulation, time of cut post-harvest stubble, and burning post-harvest stubble.

| year | Sample size of plots (N) | | Sample size of each field experimental plot (N) | | | | | |
| --- | --- | --- | --- | --- | --- | --- | --- | --- |
|  |  |  | Fertile tillers/m^2^  Y_1_ | Spiklets/fertile tillers  Y_2_ | Florets/spiklet  Y_3_ | Seed numbers/spiklet  Y_4_ | Seed weight^a^  Y_5_ (mg) | Seed yield  Z (kg/hm^2^) |
| 2003 | 105 | | 10 | 51 | 27 | 24 | 10 | 4 |
| Total sample size(n) | | | 1050 | 5355 | 2835 | 2520 | 1050 | 420 |
| 2004 | 129 | | 10 | 30 | 30 | 30 | 10 | 4 |
| Total sample size(n) | | | 1290 | 3870 | 3870 | 3870 | 1290 | 516 |
| 2005 | | 146 | 10 | 30 | 30 | 30 | 10 | 4 |
| Total sample size(n) | | | 1460 | 4380 | 4380 | 4380 | 1460 | 584 |
| Three years totally(n) 380 | | | 3800 | 13605 | 11085 | 10770 | 3800 | 1520 |

**Supplementary Table S3.** The sample size of Y_1_ to Y_5_, Z for each field experimental plot from combination of the six groups experiments on *Pascopyrum smithii* Schreb. ^a^100-seed was take as one sample at a seed water content is at 7~10%, then 10 of the 100-seed sample in each plot were averaged to obtain one sample of seed weight (Y_5_) of the plot; the total sample size (n) of Y_5_ =10×105=1050 in 2003.

| Factor | Time-of-fertilized(X_1_) | | Irrigation(X_2_) | | Nitrogcn (X_3_) | | Phosphorus (X_4_) | | Density-manipulation(X_5_) | |
| --- | --- | --- | --- | --- | --- | --- | --- | --- | --- | --- |
| **Treat**  **Level** | Level Code | Time | Level code | irrigation volume | Level code | Applied N(kg／hm^2^) | Level code | Applied P(kg／hm^2^) | Level code | Treatment |
| **1** | 0 | Autumn | -8.4 | 0 | -5 | 0 | -4.77 | 0 | 0 | Basic number |
| **2** | 1 | Tillering stage | -4.67 | 52.78 | -3.52 | 44 | -3.37 | 31 | -1 | 1/3 |
| **3** | 2 | Booting stage | -2.86 | 78 | -2.8 | 66 | -3 | 39 | -2 | 1/2 |
| **4** | 3 | Flowering stage | -2 | 90.2 | -2.06 | 88 | -2 | 61 | -2.5 | blank |
| **5** | 4 | Filling stage | -1.94 | 91 | -2 | 90 | -1.97 | 62 |  |  |
| **6** |  |  | -1.05 | 104.1 | -1.628 | 100 | -1.91 | 63 |  |  |
| **7** |  |  | -1 | 104.7 | -1.45 | 107 | -1.26 | 77 |  |  |
| **8** |  |  | 0 | 119.2 | -1.32 | 110 | -1 | 83 |  |  |
| **9** |  |  | 0.75 | 130 | -1 | 120 | -0.68 | 90 |  |  |
| **10** |  |  | 1 | 133.6 | -0.59 | 132 | -0.56 | 93 |  |  |
| **11** |  |  | 2 | 148.1 | 0 | 150 | 0 | 105 |  |  |
| **12** |  |  |  |  | 0.1 | 153 | 0.84 | 124 |  |  |
| **13** |  |  |  |  | 0.88 | 176 | 1 | 127 |  |  |
| **14** |  |  |  |  | 1 | 180 | 2 | 149 |  |  |
| **15** |  |  |  |  | 1.628 | 201 | 2.84 | 167 |  |  |
| **16** |  |  |  |  | 2 | 210 | 6.14 | 240 |  |  |
| **17** |  |  |  |  | 6.15 | 335 |  |  |  |  |
| **18** |  |  |  |  | 11 | 480 |  |  |  |  |

**Supplementary Table S4.** Coding value of X_1_to X_5_ and its corresponding usage combining the six experimental designs. 1/2 means remove the 1/2 line at tillering stage; 1/3 means remove the 1/3 line at tillering stage.

| Treatments  No. of block | Array 1  A | Array 2  B | Array 3  C | Array 4  D | Array 5  E |
| --- | --- | --- | --- | --- | --- |
| 1 | 1 | 1 | 1 | 1 | 1 |
| 2 | 1 | 2 | 2 | 2 | 2 |
| 3 | 2 | 1 | 1 | 2 | 2 |
| 4 | 2 | 2 | 2 | 1 | 1 |
| 5 | 3 | 1 | 2 | 1 | 2 |
| 6 | 3 | 2 | 1 | 2 | 1 |
| 7 | 4 | 1 | 2 | 2 | 1 |
| 8 | 4 | 2 | 1 | 1 | 2 |

**Supplementary Table S5.** Compound Matrix of L_8_（4×2^4^）Orthogonal Design. Note: 4 repeat, 32 blocks total.

| Treatment  No. of Blocks | Factor X_3_ (Nitrogen, N) | | Factor X_4_ (Phosphorus, P_2_O_5_) | |
| --- | --- | --- | --- | --- |
|  | Level Code Applied N (kg/ha) | | Level Code Applied P_2_O_5_ (kg/ha) | |
| 1, (7,13) | -1 | 0 | -1 | 0 |
| 2, (8,14) | 1 | 153 | -1 | 0 |
| 3, (9,15) | -1 | 0 | 1 | 90 |
| 4, (10,16) | -0.1315 | 66 | -0.1315 | 39 |
| 5, (11,17) | 1 | 153 | 0.3945 | 63 |
| 6, (12,18) | 0.3945 | 106.5 | 1 | 90 |

**Supplementary Table S6.** A. 2-D optimum design (1) (Nitrogen and Phosphorus). Three repeat, 18 blocks total, each with a 28 m^2^ area.

| Treatment  No. of Blocks | Factor X_3_ (Nitrogen, N) | | Factor X_4_ (Phosphorus, P_2_O_5_) | |
| --- | --- | --- | --- | --- |
|  | Level Code Applied N(kg/ha) | | Level Code Applied P_2_O_5_(kg/ha) | |
| 1 | -1 | 0 | -1 | 0 |
| 2 | 1 | 480 | -1 | 0 |
| 3 | -1 | 0 | 1 | 240 |
| 4 | -0.1315 | 208.9 | -0.1315 | 104 |
| 5 | 1 | 480 | 0.3945 | 167 |
| 6 | 0.3945 | 334.5 | 1 | 240 |

**Supplementary Table S7.** B. 2-D-optimum design (2) (Nitrogen and Phosphorus). Six blocks total, each with a 28 m^2^ area.

| Experimental Factor | Delta (∆) | (r = 2) Level-code-of-orthogonal design (r = 2)  –2 –1 0 1 2 | | | | |
| --- | --- | --- | --- | --- | --- | --- |
| Time of fertilization*(X_1_) | 1 | 1 | 2 | 3 | 4 | 5 |
| Irrigation-of-each-time (X_2_) | 14.4 (mm) | 90.4 | 104.8 | 119.2 | 133.6 | 148 |
| Applied N (X_3_) | 30 (kg/ha) | 90 | 120 | 150 | 180 | 210 |
| Applied-P_2_O_5_ (X_4_) | 22 (kg/ha) | 61 | 83 | 105 | 127 | 149 |
| Density manipulation (X_5_) | shoots/ha | 1/2 BD | 2/3 BD | BD | BD | BD |

**Supplementary Table S8.** C. Unique-factor orthogonal design. The level codes for time of fertilization: 1, previous autumn; 2, tillering time; 3, time of stem elongation; 4, time of anthesis; 5, time of grain filling. Total of 36 blocks, each with a 28 m^2^ area. BD, Basic Density.

| Treatments | X_0_ | X_1_ | X_2_ | X_3_ | X_4_ | X_5_ |
| --- | --- | --- | --- | --- | --- | --- |
| 1 | 1 | -1 | -1 | -1 | -1 | 1 |
| 2 | 1 | 1 | -1 | -1 | -1 | -1 |
| 3 | 1 | -1 | 1 | -1 | -1 | 1 |
| 4 | 1 | 1 | 1 | -1 | -1 | -1 |
| 5 | 1 | -1 | -1 | 1 | 1 | 1 |
| 6 | 1 | 1 | -1 | 1 | 1 | -1 |
| 7 | 1 | -1 | 1 | 1 | 1 | 1 |
| 8 | 1 | 1 | 1 | 1 | 1 | -1 |
| 9 | 1 | -1 | -1 | 1 | -1 | 1 |
| 10 | 1 | 1 | -1 | 1 | -1 | -1 |
| 11 | 1 | -1 | 1 | 1 | -1 | 1 |
| 12 | 1 | 1 | 1 | 1 | -1 | -1 |
| 13 | 1 | -1 | -1 | -1 | 1 | 1 |
| 14 | 1 | 1 | -1 | -1 | 1 | -1 |
| 15 | 1 | -1 | 1 | -1 | 1 | 1 |
| 16 | 1 | 1 | 1 | -1 | 1 | -1 |
| 17 | 1 | 0 | 0 | 0 | 0 | 0 |
| 18 | 1 | 0 | 0 | 0 | 0 | 0 |
| 19 | 1 | 0 | 0 | 0 | 0 | 0 |
| 20 | 1 | 0 | 0 | 0 | 0 | 0 |
| 21 | 1 | 0 | 0 | 0 | 0 | 0 |
| 22 | 1 | 0 | 0 | 0 | 0 | 0 |
| 23 | 1 | 0 | 0 | 0 | 0 | 0 |
| 24 | 1 | 0 | 0 | 0 | 0 | 0 |
| 25 | 1 | 0 | 0 | 0 | 0 | 0 |
| 26 | 1 | 0 | 0 | 0 | 0 | 0 |
| 27 | 1 | 2 | 0 | 0 | 0 | 0 |
| 28 | 1 | -2 | 0 | 0 | 0 | 0 |
| 29 | 1 | 0 | 2 | 0 | 0 | 0 |
| 30 | 1 | 0 | -2 | 0 | 0 | 0 |
| 31 | 1 | 0 | 0 | 2 | 0 | 0 |
| 32 | 1 | 0 | 0 | -2 | 0 | 0 |
| 33 | 1 | 0 | 0 | 0 | 2 | 0 |
| 34 | 1 | 0 | 0 | 0 | -2 | 0 |
| 35 | 1 | 0 | 0 | 0 | 0 | 2 |
| 36 | 1 | 0 | 0 | 0 | 0 | -2 |

**Supplementary Table S9.** Compounding matrix of unique-factor orthogonal design.

| Experimental Factor | (∆) | Level-code-of-orthogonal design (r = 2)  –3 –1 0 1 3 | | | | |
| --- | --- | --- | --- | --- | --- | --- |
| Irrigation-of each-time (X_1_) | 15.5 (mm) | 81.7 | 112.7 | 128.2 | 143.7 | 174.7 |
| (x_2_)  Applied N+P_2_O_5_* | 37.5  (kg/ha) | 75  44.12+30.88 | 150  88.24+61.76 | 187.5  110.29+77.21 | 225  132.35+92.65 | 300  176.47+123.53 |

**Supplementary Table S10.** D. Bin-factor orthogonal contract blocks. Total of 22 blocks，each with a 28 m^2^ area. Applied fertilizer mixed according to N：P_2_O_5_ = 1：0.7.

| Treatment | Irrigation (X_1_) | Applied N+ P_2_O_5_ (X_2_) |
| --- | --- | --- |
| 1 | 3 | 3 |
| 2 | 3 | 1 |
| 3 | 3 | -1 |
| 4 | 3 | -3 |
| 5 | 1 | 3 |
| 6 | 1 | 1 |
| 7 | 1 | -1 |
| 8 | 1 | -3 |
| 9 | -1 | 3 |
| 10 | -1 | 1 |
| 11 | -1 | -1 |
| 12 | -1 | -3 |
| 13 | -3 | 3 |
| 14 | -3 | 1 |
| 15 | -3 | -1 |
| 16 | -3 | -3 |
| 17 | 0 | 0 |
| 18 | 0 | 0 |
| 19 | 0 | 0 |
| 20 | 0 | 0 |
| 21 | 0 | 0 |
| 22 | 0 | 0 |

**Supplementary Table S11.** Compound matrix of Bin-factor orthogonal contract design

| Experimental Factor | Delta (∆) | (r = 2) Level-code-of-orthogonal-design (r = 2)  –1.682 –1 0 1 1.682 | | | | |
| --- | --- | --- | --- | --- | --- | --- |
| Density manipulation (X_1_) | shoots/hm^2^ | 1/2 BD | 2/3 BD | BD | BD | BD |
| Applied N (X_2_) | 30 (kg/hm^2^) | 99.5 | 120 | 150 | 180 | 200.5 |
| Plant regulator (PP333)  Paclobutrazol (PP333)*(X_3_) | a.i.0.08  kg/ha | 0.085 | 0.14 | 0.22 | 0.30 | 0.355 |

**Supplementary Table S12.** E. Tri-factor orthogonal rotary design. Total of 23 blocks, each with a 28 m^2^ area. Paclobutrazol (PP333) was sprayed during stem elongation, twice with a week break. BD, Basic Density.

| Factor  Level | A  Irrigation time | B  Planting Density | C ^1^  Time of  fertilization | D ^2^  Time of  Cut | E  Burning  Stubble |
| --- | --- | --- | --- | --- | --- |
| 1 | September + later Winter | 1/2 | Autumn | July | Burning |
| 2 | Post-harvest | 1 | Spring of the following year | September | Not burning |
| 3 | September |  |  |  |  |
| 4 | Later Winter |  |  |  |  |

**Supplementary Table S13.** F. Unique-factor orthogonal design [L_8_(4^1^×2^4^)]. Note**：**1, Applied nitrogen 150 kg/ha，P_2_O_5_ 105 kg/ha; 2, Cutting with 25 mm stubble.

|  | Precipitation (mm) | | | Average temperature (℃) | | |
| --- | --- | --- | --- | --- | --- | --- |
| Month | 2003 | 2004 | 2005 | 2003 | 2004 | 2005 |
| Jan | 1.5 | 1.9 | 2.1 | -5.5 | -8.9 | -11.2 |
| Feb | 2.1 | 0.5 | 0 | -3.6 | -2.6 | -3.7 |
| Mar | 0.4 | 4.1 | 28.1 | 4.8 | 4.9 | 2.9 |
| Apr | 5 | 0.8 | 1.2 | 8.8 | 11.6 | 8.7 |
| May | 7.5 | 6.8 | 8.3 | 17 | 16.8 | 16.3 |
| Jun | 18.5 | 18.5 | 3.9 | 21 | 20.1 | 20.9 |
| Jul | 6.8 | 10.4 | 3.2 | 21.4 | 22.4 | 23.6 |
| Aug | 20 | 13.6 | 9.8 | 20.3 | 20.6 | 21.8 |
| Sep | 4.4 | 5.6 |  | 15.9 | 18.9 |  |
| Oct | 1.2 | 1.9 |  | 10.2 | 6.9 |  |
| Nov | 2.4 | 2.6 |  | 4.3 | 2.8 |  |
| Dec | 1.5 | 0 |  | -2.1 | 0.1 |  |

**Supplementary Table S14.** Monthly precipitation (mm) and average temperature (°C) of the China Agricultural University Grassland Research Station located in the Hexi Corridor, Jiuquan, Gansu Province from 2003 to 2005.

1. [↑](#footnote-ref-1)
